# Safety of aripiprazole for tics in children and adolescents: A systematic review and meta-analysis

**DOI:** 10.1097/MD.0000000000015816

**Published:** 2019-05-31

**Authors:** Chunsong Yang, Qiusha Yi, Lingli Zhang, Hao Cui, Jianping Mao

**Affiliations:** a Department of Pharmacy, Evidence-based Pharmacy Center, West China Second Hospital, Sichuan University; b Key Laboratory of Birth Defects and Related Diseases of Women and Children (Sichuan University), Ministry of Education; c West China School of Medicine; d Department of Pediatric Neurology, West China Hospital, Sichuan University, Chengdu; e Department of Health, Zhuhai Maternity and Child Health Hospital, Zhuhai, Guangdong, China.

**Keywords:** aripiprazole, children, safety, systematic review, tic disorders

## Abstract

**Background:**

Aripiprazole is widely used in the management of tic disorders (TDs), we aimed to assess the safety of aripiprazole for TDs in children and adolescents.

**Methods:**

A systematic literature review was performed in the databases of MEDLINE, Embase, the Cochrane Library and 4 Chinese databases, from inception to February 2019. All types of studies evaluating the safety of aripiprazole for TDs were included. The quality of studies was assessed using the Cochrane Risk of Bias tool, the Newcastle–Ottawa Scale tool, the National Institute of Clinical Excellence, the CARE (Case Report) guidelines according to types of studies. Risk ratio (RR) and incidence rate with a 95% confidence interval (CI) were used to summarize the results.

**Results:**

A total 50 studies involving 2604 children met the inclusion criteria. The result of meta-analysis of randomized controlled trials showed that there was a significant difference between aripiprazole and haloperidol with respect to rate of somnolence (RR = 0.596, 95% CI: 0.394, 0.901), extrapyramidal symptoms (RR = 0.236, 95% CI: 0.111, 0.505), tremor (RR = 0.255, 95% CI: 0.114, 0.571), constipation (RR = 0.148, 95% CI: 0.040, 0.553), and dry mouth (RR = 0.141, 95% CI: 0.046, 0.425). There was a significant difference between aripiprazole and placebo in the incidence rate of adverse events (AEs) for somnolence (RR = 6.565, 95% CI: 1.270, 33.945). The meta-analysis of incidence of AEs related to aripiprazole for case series studies revealed that the incidence of sedation was 26.9% (95% CI: 16.3%, 44.4%), irritability 25% (95% CI: 9.4%, 66.6%), restlessness 31.3% (95% CI: 13%, 75.1%), nausea and vomiting 28.9% (95% CI: 21.1%, 39.5%), and weight gain 31.3% (95% CI: 10.7%, 91.3%).

**Conclusion:**

Aripiprazole was generally well tolerated in children and adolescents. Common AEs were somnolence, headache, sedation, nausea, and vomiting. Further high-quality studies are needed to confirm the safety of aripiprazole for children and adolescents with TDs.

## Abstract 2

Supplemental Digital Content is available in the text

## 1 Introduction

Tic disorders (TDs) are very common neurodevelopmental condition in children and adolescents^[1]^ and are characterized by the presence of abrupt and repeated motor movements or vocalization. There are 3 kinds of TD: transient tic, chronic tic, and Tourette syndrome with prevalence rates 2.99%, 1.61%, and 0.77%, respectively.^[2]^ In general, the severity of TDs wanes in late adolescence, and the prevalence rates of TDs in adulthood become much lower. There are various comorbid psychiatric conditions with TDs, such as obsessive–compulsive disorder (OCD), attention-deficit hyperactivity disorder (ADHD), and sustained social problems. TDs and these comorbidities are associated with many serious impairments in social functioning as well as emotional and educational impairment, which can have serious negative impacts on quality of life.^[3–5]^

Currently, pharmacological treatment is the most common intervention for patients with TDs, including typical antipsychotics (e.g., haloperidol, pimozide), atypical antipsychotics (e.g., risperidone, quetiapine), analgesics (e.g., naltrexone, propoxyphene), anticonvulsants (e.g., topiramate), antidepressants (e.g., desipramine), among others. However, the use of these treatments is associated with several adverse events (AEs), including tardive dyskinesia, extrapyramidal syndrome, and electrocardiographic abnormality.^[6,7]^

Aripiprazole, a dopamine agonist and 5-HT1A receptor, could act as a dopamine D2 partial agonist based on local dopamine system surroundings.^[8,9]^ It is extensively used in the management of TDs in the United States, China, and other countries. Yang et al^[10]^ reviewed 12 trials including 935 participants aged between 4 and 18 years, involving aripiprazole for children with TDs. Those authors confirmed that aripiprazole appears to be a new treatment approach for children with TDs; the systematic review also pointed out that drowsiness, increased appetite, nausea, and headache were common AEs with use of aripiprazole for tics. However, that study only included randomized controlled trials (RCTs) and did not include a quantitative analysis of safety; therefore, the safety of aripiprazole was not well evaluated.

A considerable number of trials have researched the efficacy and safety of aripiprazole for patients with TDs; these studies have provided evidence of the comparative efficacy and safety of aripiprazole for TDs.^[11–14]^ However, several new reports have been published demonstrating that the findings for the relative safety of aripiprazole in children and adolescents need to be updated.

Therefore, to provide additional information on the safety of aripiprazole, we included all types of studies and performed a meta-analysis to assess the safety of aripiprazole for TDs in children and adolescents.

## 2 Methods

This meta-analysis was conducted strictly according to the Preferred Reporting Items for Systematic Reviews and Meta Analyses (PRISMA) guidelines, and the ethical approval and informed consent were unnecessary since the meta-analysis was aimed to summarize the previous studies.

### 2.1 Search strategy and study selection

A systematic literature review was performed in the databases of MEDLINE, Embase, the Cochrane Library, the Chinese Biomedical Literature Database, China Knowledge Resource Integrated Database, VIP Database, and Wanfang Database, from inception to March 2018. Citations of relevant studies were searched for appropriate articles as well. The search terms included “aripiprazole,” “Tourette syndrome,” “tic disorders,” and “tics.” According to the specific requirements of the database, the terms were combined into different retrieval expressions. (See Supplemental Digital Content, which illustrates search strategy for Each Database).

### 2.2 Inclusion and exclusion criteria

The inclusion criteria were developed using the PICOS (P: population; I: intervention; C: comparison; O: outcome; S: study design) framework, as follows:

1. Population: aged <18 years old, with a clinical diagnosis of TD
2. Intervention: aripiprazole
3. Comparison: placebo or other types of pharmacotherapies
4. Outcome: prevalence rate of all types of AE
5. Study design: all types of studies, including RCT, non-RCT, cohort study, case-control study, case series study, and case report, with data extraction and quality assessment

Meanwhile, we restricted the language of publications English or Chinese. Through reading the title, abstract, and full text, we judged whether studies met the inclusion criteria.

Data were extracted by 1 author and checked by another author using an Excel form, which included the following information: study information, age, sex, intervention, control, treatment period, time of follow-up, diagnostic criteria, and prevalence rate of AEs.

The quality of all RCTs and non-RCTs was assessed using the Cochrane Risk of Bias tool according to the Cochrane Handbook for Systematic Reviews of Interventions (www.cochranehandbook.org): Random sequence generation; Allocation concealment; Blinding of participants; Blinding of outcome assessment; Incomplete outcome data; Selective reporting; Other sources of bias.^[15]^ The qualities of case-control studies and cohort studies were assessed using the Newcastle–Ottawa Scale tool.^[16]^ Assessment of risk of bias in case series was based on the recommendations of the National Institute of Clinical Excellence (NICE): Case series in more than 1 center, that is, multicenter study; Is the hypothesis/aim/objective of the study clearly described? Are the inclusion and exclusion criteria (case definition) clearly reported? Is there a clear definition of the outcomes reported? Were data collected prospectively? Is there an explicit statement that patients were recruited consecutively? Are the main findings of the study clearly described? Are outcomes stratified? (e.g., by disease stage, abnormal test results, patient characteristics).^[17]^ Quality appraisal of case reports was conducted according to the CARE (CAse REport) guidelines: Title; Keywords; Abstract; Introduction; Patient Information; Clinical Findings; Timeline; Diagnostic Assessment; Therapeutic Intervention; Follow-up and Outcomes; Discussion; Patient Perspective; Informed Consent.^[18]^

### 2.3 Statistical analysis

All statistical analyses were performed using Stata 12.0 (StataCorp, College Station, TX). Risk ratio (RR) and incidence rate with a 95% confidence interval (CI) were used to summarize the results. The significance of evidence was evaluated using the Z-test. We used the Q test and I2 statistic to assess the percentage of heterogeneity.^[19]^ When the outcome of the Q test was *P* < .1 and I^2^ > 50%, revealing the significance of heterogeneity, then a random-effects model was applied to evaluate the summary results; otherwise, a fixed-effects model was applied. Sensitivity analysis was performed on a network excluding trials with low quality. Funnel plots were used to evaluate publication bias, if the number of included studies for 1 outcome was 10 or more.

## 3 Results

### 3.1 Included studies

**Figure 1:**
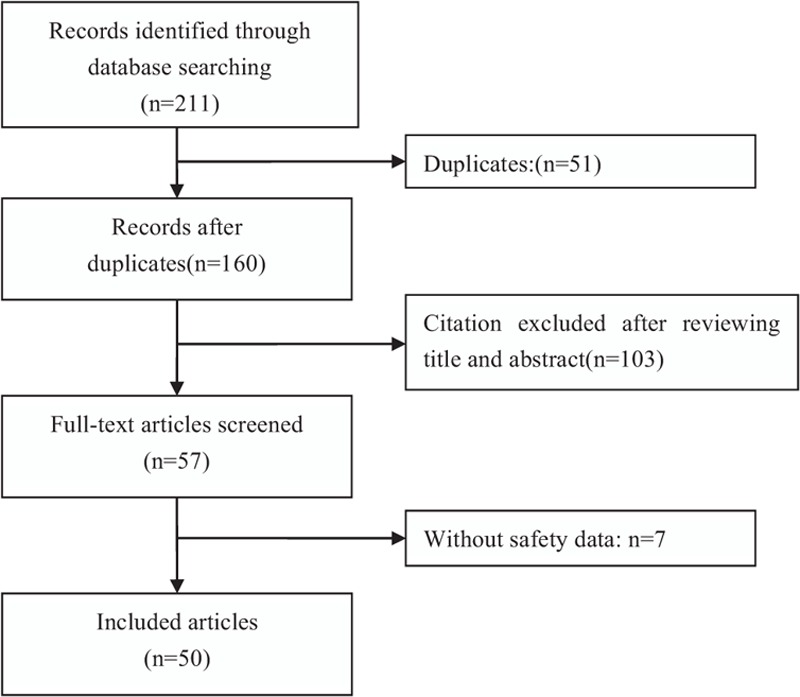
Flow chart of literature screening and the selection process.

Our initial database search yielded 211 studies. After reading the title, abstract, and full text, 50 studies met the inclusion criteria (Fig. 1); Of these, 24 were English articles and 26 were Chinese articles, involving a total of 2604 children with TDs. The characteristics of included studies are depicted in Table 1  .

**Table 1:**
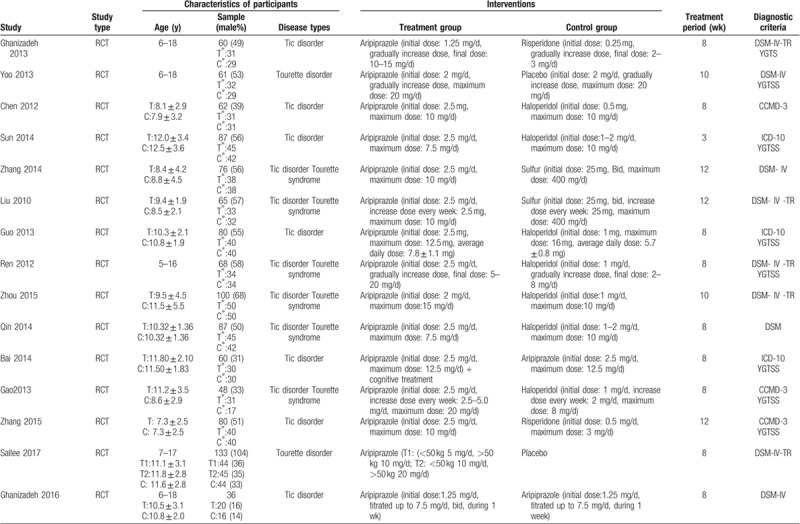
General characteristics of included RCTs.

**Table 1 (Continued):**
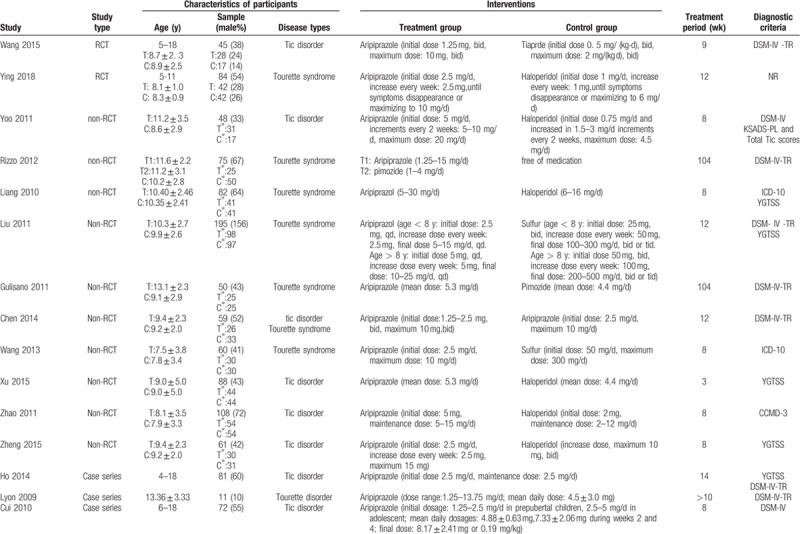
General characteristics of included RCTs.

**Table 1 (Continued):**
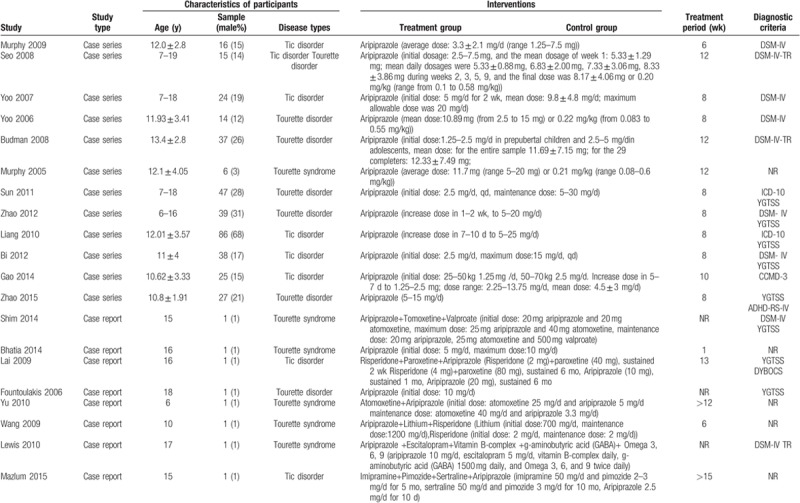
General characteristics of included RCTs.

A total 17 RCTs^[20–39]^ were included in our review, involving 1232 participants aged 0 to 18 years. The period of treatment ranged from 4 to 12 weeks. Thirteen studies were conducted in China, 2 in Iran, 1 in South Korea, and 1 multicenter trial conducted in the United States, Canada, Hungary, and Italy.

In terms of case-control studies, a total 10 non-RCT^[40–44]^ articles were eligible for inclusion. The included studies involved 826 children under the age of 18 years, with a treatment duration of 8 to 104 weeks. Seven were conducted in China, 1 in Italy, 1 in South Korea, and 1 in the United States.

In terms of case series, we identified a total of 15 studies^[45–60]^ on the safety of aripiprazole in the treatment of Tourette syndrome, a total 538 children. Eight studies were conducted in China, 4 in the United States, and 3 in South Korea.

Eight case reports^[61–68]^ were included in our review, with a total of 8 children. Three studies were carried out in China; the remaining 5 studies were conducted in South Korea, the United States, Greece, Turkey, and India, respectively.

### 3.2 Quality assessment

To assess the methodological quality of RCTs, only 9 studies (52.9%) used an adequate method of random sequence generation; the remaining studies did not mention any method or used an inappropriate allocation method. Three studies (17.6%) implemented allocation concealment. Similarly, blinding of participants and outcome assessment were not specified; 3 studies (17.6%) described blinding of participants and outcome assessment, and 2 studies (12.5%) were judged to be prone to a high risk of bias. The risk of bias regarding incomplete outcome data was judged to be high in 1 report (6.3%). Reporting bias was not detected in any of the included studies, and no other bias was found.

In the assessment of methodology quality of non-RCTs, none of these studies described appropriate random sequence generation. Five studies (50%) described as open-label trials had adequate allocation concealment; the remaining 5 studies (50%) did not include sufficient information to evaluate this item, leading to the determination of unclear risk. For blinding, 3 studies (30%) were assessed as having high risk of bias in the blinding of participants and personnel. Similarly, 3 studies (30%) were judged to be prone to high risk of bias in the blinding of outcome assessment; the remaining studies (70%) could not be evaluated because of insufficient information. In terms of incomplete outcome data, 4 studies (40%) were described as unclear risk of bias, and the remainder (60%) showed low risk of bias. Reporting bias was not detected in any of the included studies; no other bias was found.

Case studies had a mean score of 5.67 points according to the NICE guidelines checklist. Only 2 studies were multicenter studies, and outcomes were not stratified in either study; the remaining indicators demonstrated fair good quality.

We assessed the methodological quality of case reports based on the 13 items of the CARE guidelines. All case reports described the items of title, patient information, clinical findings, time line, therapeutic interventions, follow-up and outcomes, and discussion. Six studies included the items of introduction and diagnostic assessment, and 5 studies comprised the items keywords and abstract. Only 2 reports had a low risk of informed consent. All reports had a high risk of patient perspective. The quality assessment of the included studies is summarized in Table 2.

**Table 2:**
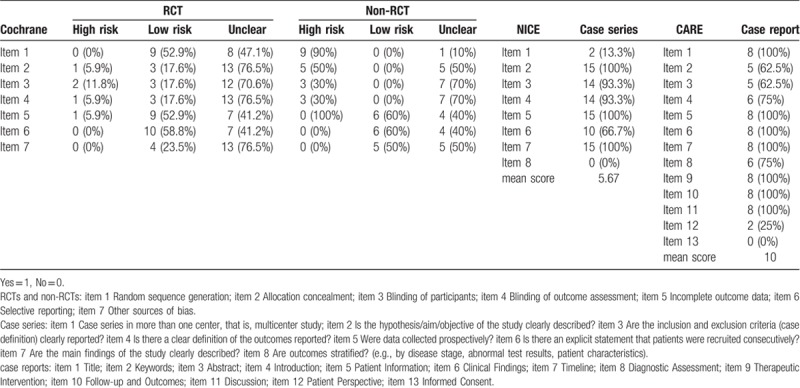
Quality assessment of includes studies.

### 3.3 RCT safety results

The most common AEs with aripiprazole in RCTs were somnolence (17.2%), increased appetite (13.5%), sedation (13.2%), dyspepsia (9.7%), and nasopharyngitis (9.1%).

#### 3.3.1 Aripiprazole versus other pharmacotherapies

##### 3.3.1.1 Neurological and psychiatric symptoms

We compared aripiprazole with haloperidol, risperidone, and sulfur with respect to various types of neurological and psychiatric AEs. The results of the meta-analysis showed that there was a significant difference between aripiprazole and haloperidol in the rates of somnolence (RR = 0.596; 95% CI: 0.394, 0.901; *P* = .014), extrapyramidal symptoms (RR = 0.236; 95% CI: 0. 0.111, 0.505; *P* = .000), and tremor (RR = 0.255; 95% CI: 0.114, 0.571; *P* = .001). The differences for the remaining AEs showed no statistical significance (*P* > .05).

#### 3.3.2 Digestive system

The included studies reported that the occurrence of gastrointestinal AEs with aripiprazole was significantly lower than those with haloperidol for constipation (RR = 0.148; 95% CI: 0.040, 0.553; *P* = .004). Although the rate of AEs with use of aripiprazole with respect to the digestive system was lower than those with use of risperidone and sulfur, there was no statistical difference (*P* > .05).

#### 3.3.3 Cardiovascular system

Four types of AEs of the cardiovascular system (abnormal electrocardiogram, chest discomfort, tachycardia, bradycardia) were reported among the aripiprazole and haloperidol groups. Nevertheless, the differences were not statistically significant (*P* > .05).

#### 3.3.4 Urinary system

Only 2 studies reported AEs affecting the urinary system. There were no urinary AEs with aripiprazole; the use of sulfur had 1 reported case of urinary AEs. Nocturia occurred with risperidone in 4 cases; however, there were no significant differences (*P* > .05).

#### 3.3.5 Respiratory system

The included studies reported that the occurrence of nasopharyngitis with aripiprazole was significantly lower than that with placebo (*P* < .05). As for upper respiratory infection, we found no significant differences.

#### 3.3.6 Other AEs

Meta-analysis of 1 study (n = 60) that compared the occurrence of blurred vision and itching between aripiprazole and risperidone showed that there were differences, but without statistical significance (*P* > .05). A significant difference was observed in the incidence rate of dry mouth between aripiprazole and haloperidol treatment groups (RR = 0.141; 95% CI: 0.046, 0.425; *P* = .001).

#### 3.3.7 Aripiprazole versus placebo

We retrieved 2 RCTs (n = 194) that reported AEs in a positive control group and placebo group. The results of meta-analysis showed that there was no significant difference (*P* > .05) in the incidence rate of AEs between aripiprazole and placebo, except for somnolence (RR = 6.565; 95% CI: 1.270, 33.945; *P* = .025), as shown in Table 3  .

**Table 3:**
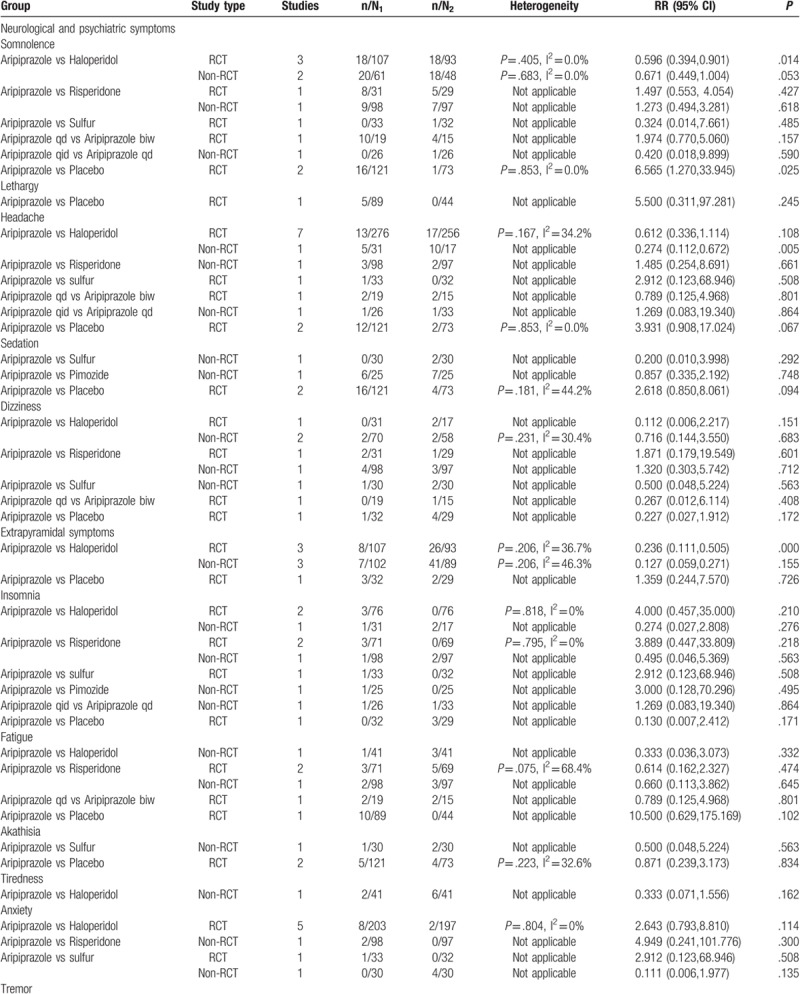
Meta-analysis of RCT and non-RCT.

**Table 3 (Continued):**
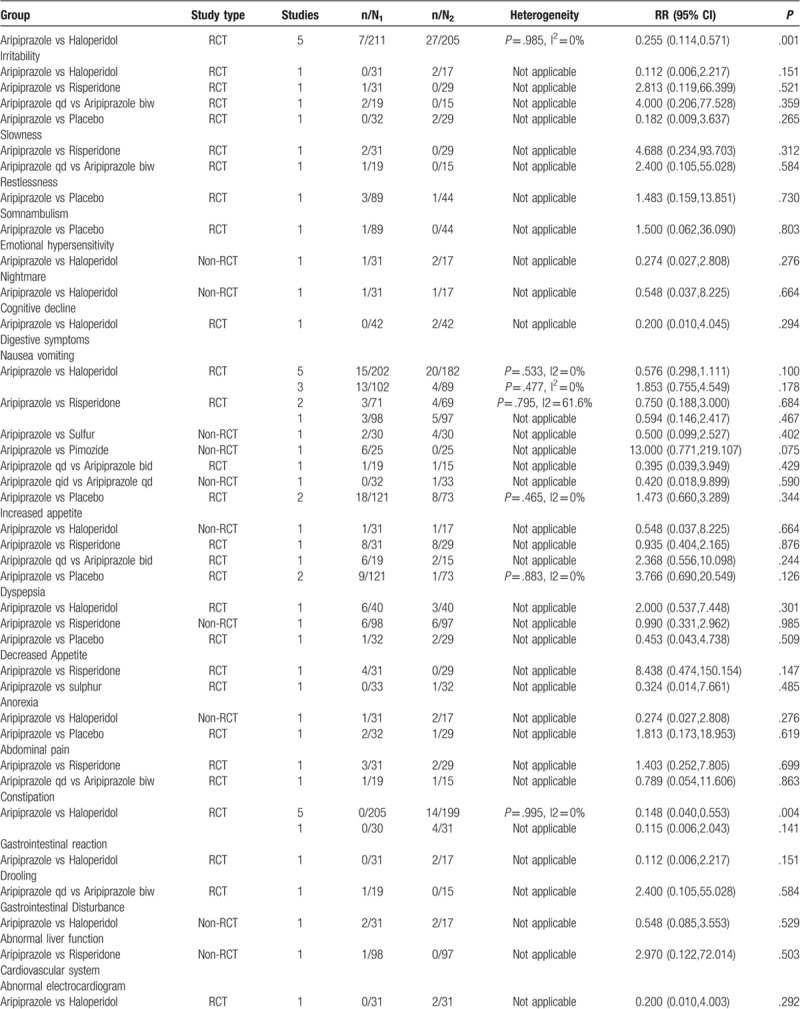
Meta-analysis of RCT and non-RCT.

**Table 3 (Continued):**
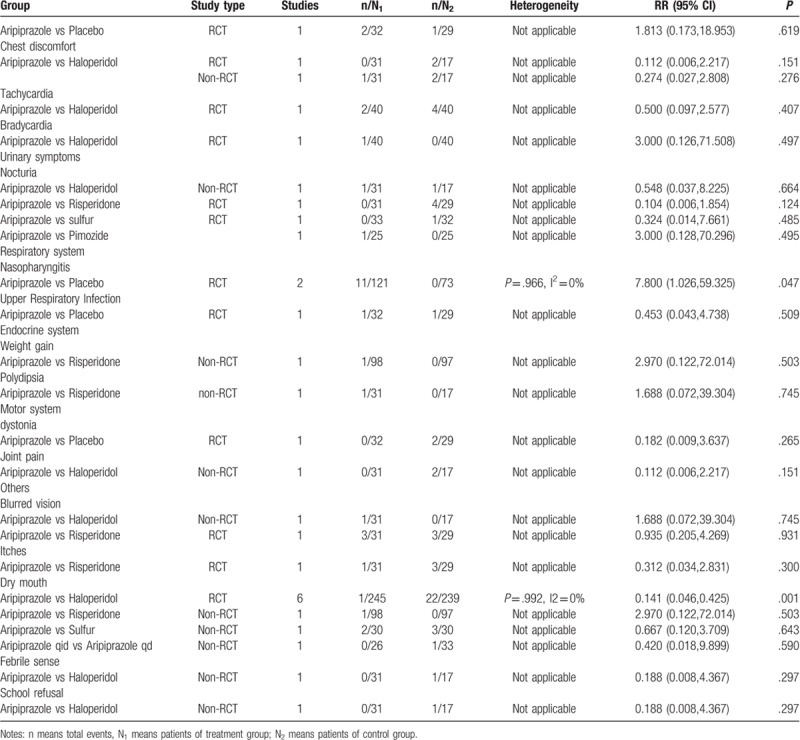
Meta-analysis of RCT and non-RCT.

### 3.4 Non-RCT safety results

We compared aripiprazole with other pharmacotherapies with respect to safety in individual human systems. The most common AEs with aripiprazole in non-RCTs were somnolence (15.7%), sedation (10.9%), nausea and vomiting (8.4%), extrapyramidal symptoms (6.9%), and gastrointestinal disturbance (6.4%).

The results of meta-analysis revealed that there was no significant difference in the rate of AEs between aripiprazole and haloperidol, risperidone, sulfur, and pimozide. Similar statistical differences were found for the incidence of AEs between 2 aripiprazole treatment groups with different administration frequency (aripiprazole q.i.d. vs aripiprazole q.d.), as shown in Table 3  .

### 3.5 Case series safety results

There were 13 studies describing AEs in detail whereas the other 2 only briefly mentioned AEs. The most common incidence of AEs with use of aripiprazole was sedation (26.9%; 95% CI: 16.3%, 44.4%), irritability (25%; 95% CI: 9.4%, 66.6%), restlessness (31.3%; 95% CI: 13%, 75.1%), nausea and vomiting (28.9%; 95% CI: 21.1%, 39.5%), and weight gain (31.3%; 95% CI: 10.7%, 91.3%) (*P* < .05). There were no significant differences for tiredness; stomach discomfort; or muscle, bone, or joint pain/conditions (*P* > .05) (Table 4).

**Table 4:**
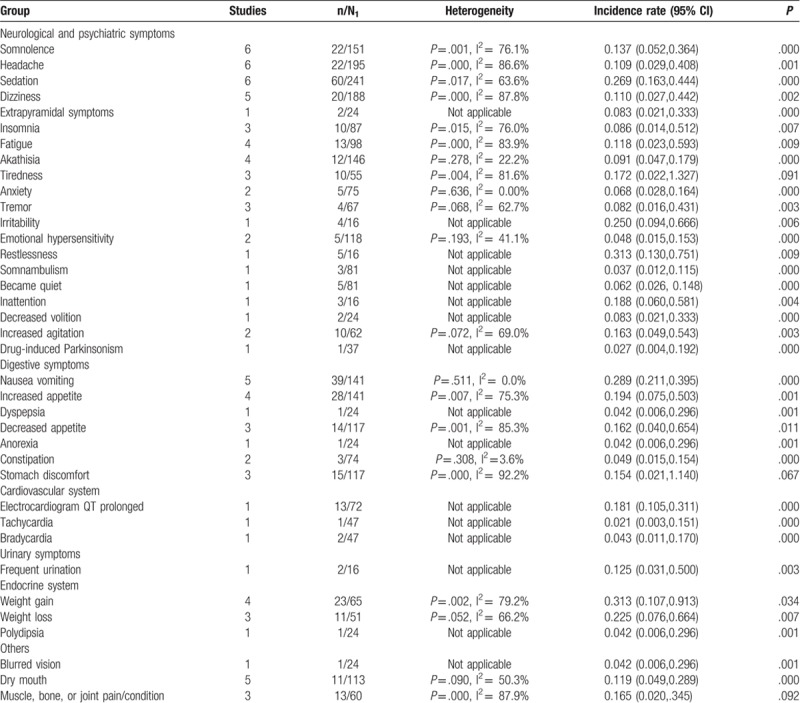
Meta-analysis of case series.

### 3.6 Case report safety results

Five of 8 cases (62.5%) mentioned or described AEs, which included convulsions, mania, fidgeting, trembling, inarticulate speech, slow motion, dizziness, muscle cramps, nystagmus, torticollis, and insomnia.

### 3.7 Sensitivity analysis

In regard to the primary outcome, after excluding trials with low-quality RCTs which did not report appropriate randomized method and allocation, no material change of the pooled estimated effects in sensitivity analysis was found (Table 5). The minor change of estimated effects between interventions was as follows: Aripiprazole versus Placebo (Somnolence).

**Table 5:**
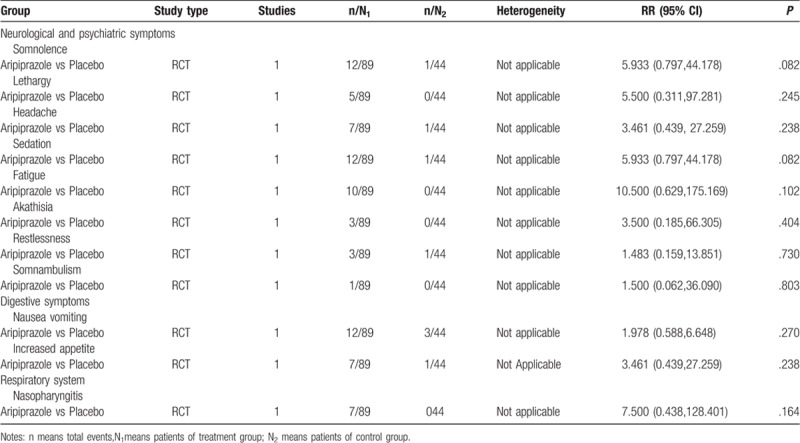
Meta-analysis of high-quality RCT.

### 3.8 Publication bias

Finally, funnel plots were not used because the number of included studies in 1 comparison had insufficient statistical power, according to the recommendations of the Cochrane Handbook for Systematic Reviews of Interventions.

## 4 Discussion

In this study, we evaluated the safety of aripiprazole for TDs in the wider context. To our knowledge, this is the first and most comprehensive meta-analysis of this topic. Our analyses were based on 50 studies (17 RCT, 10 case control, 8 case report, 15 case series) including 2604 children with TDs.

Results from the meta-analysis showed that the rate of AEs with aripiprazole was significantly lower than those with haloperidol in some fields. In terms of neurological and psychiatric symptoms, only the comparison of aripiprazole with haloperidol and aripiprazole with placebo showed a significant difference in RCTs; the other studies showed nonsignificant differences. In terms of AEs of the digestive system, only the comparison of aripiprazole and haloperidol showed a significant difference in RCTs. In terms of respiratory system AEs, a significant difference was found only between aripiprazole and placebo in RCTs; other studies showed a nonsignificant difference. In terms of AEs of the cardiovascular, urinary, and motor systems, we found nonsignificant differences between aripiprazole and other pharmacotherapies. Overall, the results of our systematic review favored the clinical use of aripiprazole, which can be considered an excellent treatment option for TDs as aripiprazole shows good tolerability in children and adolescents. Our findings agreed with those of previous relevant studies. Considering that the quality of studies included here was poor, it is necessary to confirm our findings in future studies.

There are some strengths that should be noted in our meta-analysis. First, this study is based on the PRISMA reporting recommendations.^[69]^ Second, to ensure the coverage of all relevant AEs, a comprehensive search of the literature was conducted in which we included any type of study, to reduce the possibility of publication bias. Third, 2 independent authors were involved in the phases of study retrieval, data extraction, and quality assessment. In addition, another author checked the consistency of the results and resolved disagreements. Fourth, the tools used in this review to assess the risk of bias are the most widely used and accepted.

Several important limitations of this review also emerged. First, although the report retrieval was comprehensive, it is still possible that unpublished reports were not found. In addition, we failed to search several websites of special agencies that report adverse drug events. Second, some of our results focused on short-term outcomes, which cannot be generalized to long-term safety. Third, the measures and definition of some AEs might differ among the included studies, which might cause clinical heterogeneity. Fourth, no protocol was established before the study was carried out. Fifth, we could not combine data from different dose arm. It is difficult to separate different dose arm, because every study gave the appropriate dose for patients according to the weight and age.

## 5 Conclusion

In conclusion, we found that aripiprazole had clinically relevant tolerability in children and adolescents. Aripiprazole might be viewed as an important treatment option for patients with TDs in these age groups. The common AEs were somnolence, headache, sedation, and nausea and vomiting. There is a need for further studies to confirm the use of aripiprazole in children and adolescents with TDs.

## Acknowledgments

The authors thank Group of People with Highest Risk of Drug Exposure of International Network for the Rational Use of Drugs, China for providing support to coordinate circulation of the manuscript to all co-authors and collect comments from all coauthors.

## Author contributions

YCS, YQS, ZLL, CH, and MJP contributed to planning, supervision, writing, and analysis of the study; YCS, YQS, and ZLL independently selected titles, abstract and full text; YCS, YQS, CH, and MJP each contributed to data collection, writing the manuscript and review of the literature. All authors have read and approved the final manuscript.

**Conceptualization:** Jianping Mao.

**Methodology:** Chunsong Yang, Lingli Zhang, Hao Cui.

**Software:** Jianping Mao.

**Validation:** Hao Cui.

**Visualization:** chunsong yang.

**Writing – original draft:** chunsong yang, qiusha yi.

**Writing – review & editing:** chunsong yang.

## Supplementary Material

Supplemental Digital Content
