# Supplementary material for: Safety of aripiprazole for tics in children and adolescents: A systematic review and meta-analysis
Source: Medicine (Baltimore). 2019 May 31;98(22):e15816. doi: 10.1097/MD.0000000000015816 (PMC6709053; doi:10.1097/MD.0000000000015816)
Supplement: Supplemental Digital Content [file medi-98-e15816-s001.docx]

**Supplemental Digital Content: Search Strategy for Each Database**

**MEDLINE(Ovid)**

#1 tic disorders.mp. or Tic Disorders/

#2 tic.mp. or Tics/

#3 Tourette syndrome.mp. or Tourette Syndrome/

#4 aripiprazole.mp. or Aripiprazole/

#5 #1 or #2 or #3

#6 #4 and #5

**Embase (Ovid)**

#1 tic/ OR tics.mp.

#2 tourette syndrome.mp.

#3 Gilles de la Tourette syndrome/

#4 tic disorders.mp.

#5 aripiprazole.mp. or aripiprazole/

#6 #1 or #2 or #3 or #4

#7 #5 and #6

**Cochrane library**

#1 tics d:ti,ab,kw

#2 tourette syndrome:ti,ab,kw

#3 Tourette syndrome:ti,ab,kw

#4 #1 or #2

#5 #3 and #4

#1 (Tourette syndrome):ti,ab,kw

#2 (tic disorders):ti,ab,kw

#3 (tics):ti,ab,kw

#4 MeSH descriptor: [Tics] explode all trees

#5 MeSH descriptor: [Tic Disorders] explode all trees

#6 MeSH descriptor: [Tics] explode all trees

#7 MeSH descriptor: [Aripiprazole] explode all trees

#8 (aripiprazole):ti,ab,kw

#9 #1 or #2 or #3 or #4 or #5 or #6

#10 #7 or #8

#11 #9 and #10

**CNKI**

SU=(' tic disorders'+' Tourette syndrome')*' Aripiprazole '

**VIP**

（M= tic disorders +M= Tourette syndrome） AND M= Aripiprazole

**WANGFANG**

（Subject Term:“tic disorders”+ Subject Term:“Tourette syndrome”）* Subject Term:“Aripiprazole”

**CBM:**

(Default [smart]: tic disorders or Default [smart]: tic disorders) and Default [smart]: Aripiprazole
